# Noninvasive Assessment of Atrial Fibrillation Complexity in Relation to Ablation Characteristics and Outcome

**DOI:** 10.3389/fphys.2018.00929

**Published:** 2018-07-17

**Authors:** Marianna Meo, Thomas Pambrun, Nicolas Derval, Carole Dumas-Pomier, Stéphane Puyo, Josselin Duchâteau, Pierre Jaïs, Mélèze Hocini, Michel Haïssaguerre, Rémi Dubois

**Affiliations:** ^1^Institute of Electrophysiology and Heart Modeling (IHU Liryc), Foundation Bordeaux University, Pessac-Bordeaux, France; ^2^University of Bordeaux, CRCTB, U1045, Bordeaux, France; ^3^INSERM, CRCTB, U1045, Bordeaux, France; ^4^Bordeaux University Hospital Centre Hospitalier Universitaire, Electrophysiology and Ablation Unit, Pessac, France; ^5^CardioInsight, Medtronic, Minneapolis, MN, United States

**Keywords:** atrial fibrillation, catheter ablation, body surface potential maps, principal component analysis, atrial fibrillation complexity

## Abstract

**Background:** The use of surface recordings to assess atrial fibrillation (AF) complexity is still limited in clinical practice. We propose a noninvasive tool to quantify AF complexity from body surface potential maps (BSPMs) that could be used to choose patients who are eligible for AF ablation and assess therapy impact.

**Methods:** BSPMs (mean duration: 7 ± 4 s) were recorded with a 252-lead vest in 97 persistent AF patients (80 male, 64 ± 11 years, duration 9.6 ± 10.4 months) before undergoing catheter ablation. Baseline cycle length (CL) was measured in the left atrial appendage. The procedural endpoint was AF termination. The ablation strategy impact was defined in terms of number of regions ablated, radiofrequency delivery time to achieve AF termination, and acute outcome. The atrial fibrillatory wave signal extracted from BSPMs was divided in 0.5-s consecutive segments, each projected on a 3D subspace determined through principal component analysis (PCA) in the current frame. We introduced the nondipolar component index (NDI) that quantifies the fraction of energy retained after subtracting an equivalent PCA dipolar approximation of heart electrical activity. AF complexity was assessed by the NDI averaged over the entire recording and compared to ablation strategy.

**Results:** AF terminated in 77 patients (79%), whose baseline AF CL was 177 ± 40 ms, whereas it was 157 ± 26 ms in patients with unsuccessful ablation outcome (*p* = 0.0586). Mean radiofrequency emission duration was 35 ± 21 min; 4 ± 2 regions were targeted. Long-lasting AF patients (≥12 months) exhibited higher complexity, with higher NDI values (≥12 months: 0.12 ± 0.04 vs. <12 months: 0.09 ± 0.03, *p* < 0.01) and short CLs (<160 ms: 0.12 ± 0.03 vs. between 160 and 180 ms: 0.10 ± 0.03 vs. >180 ms: 0.09 ± 0.03, *p* < 0.01). More organized AF as measured by lower NDI was associated with successful ablation outcome (termination: 0.10 ± 0.03 vs. no termination: 0.12 ± 0.04, *p* < 0.01), shorter procedures (<30 min: 0.09 ± 0.04 vs. ≥30 min: 0.11 ± 0.03, *p* < 0.001) and fewer ablation targets (<4: 0.09 ± 0.03 vs. ≥4: 0.11 ± 0.04, *p* < 0.01).

**Conclusions:** AF complexity can be noninvasively quantified by PCA in BSPMs and correlates with ablation outcome and AF pathophysiology.

## Introduction

Atrial fibrillation (AF) is the most common cardiac arrhythmia, and it is associated with an increased risk of stroke, heart failure, and mortality (Kirchhof et al., 2016). Despite the apparently random and uncoordinated electrical wavefronts propagating through the atria (Moe, 1962), several studies have confirmed the presence of intrinsic organization of atrial activations during AF, whose triggering and maintenance may be explained by some underlying, deterministic mechanisms (Schricker et al., 2014), involving multiple atrial wavelets and re-entrant sources (Allessie et al., 1977; Konings et al., 1994; Pandit and Jalife, 2013; Haissaguerre et al., 2014). Complexity of the atrial substrate is strictly correlated with the evolutionary nature of AF, and it tends to increase in more severe, persistent forms of this disease (Wijffels et al., 1995). Despite the increasing use of catheter ablation (CA) to treat persistent and chronic AF patients, its results are not satisfactory yet and extremely disparate due to the variety of ablation approaches currently adopted (Verma et al., 2015).

Even though AF electrophysiological complexity can be assessed using invasive direct contact mapping, there is an increasing interest in noninvasive methodologies as well, due to the immediate availability of cardiac body surface potentials in clinical daily practice (Lankveld et al., 2014) and their proven ability to predict the outcome of AF cardioversion or ablation and help identify positive responders to therapy. Most of the complexity ECG measures investigated so far have been determined both in the frequency [e.g., dominant frequency, DF (Bollmann et al., 2003)] and the time domain [fibrillatory wave amplitude (Nault et al., 2009; Cheng et al., 2013), sample entropy (Alcaraz et al., 2011), AF cycle length (CL, Matsuo et al., 2009)]. Correlation between several markers of complexity from standard electrocardiogram (ECG) and invasive measures of AF complexity from high density epicardial mapping has been systematically investigated in Bonizzi et al. (2014). Spectral measures of spatiotemporal organization computed from surface ECG were able to discriminate between persistent and long-standing AF (Uldry et al., 2012). Atrial complexity indices from ECG could also predict sinus rhythm (SR) maintenance in patients undergoing electrical cardioversion, either alone (Lankveld et al., 2016a) or in combination with other clinical parameters (Zeemering et al., 2017). An optimal set of ECG descriptors of AF complexity has also been determined in Lankveld et al. (2016b), and it was shown to be predictive of CA outcome.

The main limitation of the aforementioned methods is that most of them were applied to single or pairs of ECG leads [typically V_1_, exhibiting the highest atrial-to-ventricular amplitude ratio (Petrutiu et al., 2006), or the precordial leads], thus the spatial diversity of multilead recordings was not fully exploited. Furthermore, frequency domain measures of AF complexity may be inaccurate if they are assessed in short ECG recordings or if QRST cancelation is not properly performed. This background justifies the interest in exploiting the spatial diversity of multilead recordings to assess the complexity of the AF wavefront propagation.

A multilead characterization of AF spatiotemporal organization in body surface potential maps (BSPMs) has been proposed in Bonizzi et al. (2010), where it was quantified as a function of the error of signal estimation by principal component analysis (PCA). Despite the relevance of these results and the proven superiority of this methodology over standard single-lead analysis, its ability to guide AF therapy and its applicability to a real clinical scenario were not verified in that study. In Di Marco et al. (2012), AF spatial complexity was defined in terms of the residual cumulative variance of the three dominant PCA sources and correlated with its spectral variability overs BSPM electrodes. However, body surface cardiac activity characterization has not been correlated with the properties of the underlying atrial substrate nor correlated with AF treatment strategy. Multilead measures of atrial signal amplitude (Meo et al., 2013a) and spatiotemporal variability (Meo et al., 2013b) obtained by PCA proved to be predictive of CA outcome. Nevertheless, the lack of comparison with intracardiac recordings hampered their validation as indices of AF complexity.

This study takes a step from this research and puts forward a noninvasive PCA-based approach for the quantification of AF spatiotemporal complexity. Additionally, in Meo et al. (2017) some PCA-derived parameters were developed to predict changes in body surface complexity during ventricular fibrillation episodes. In this study, a similar methodology is proposed to quantify the spatiotemporal organization of AF wavefront propagation pattern as measured on body surface potentials. The approach proposed not only provides some insights about AF chronification reflecting the severity of the alterations of the atrial substrate, but it also predicts CA outcome and correlates with procedural characteristics.

## Methods

### Study population

A group of 97 persistent AF patients was enrolled in this study. Their baseline characteristics are reported in Table 1.

**Table 1:** Study population characteristics.

	***n* = 97**
Sex, male, *n* (%)	80 (82)
Age, mean ± std, years	64 ± 11
Hypertension, *n* (%)	42 (42)
Diabetes mellitus, *n* (%)	10 (17)
Embolic events, *n* (%)	8 (8)
Structural heart disease, *n* (%)	61 (62)
Ischemic, *n* (%)	10 (10)
Valvular, *n* (%)	6 (6)
Hypertrophic, *n* (%)	8 (8)
Dilated, *n* (%)	35 (36)
Other, *n* (%)	2 (2)
Left ventricular ejection fraction, mean ± std, %	52 ± 13
LA diameter, parasternal long axis, mm	48 ± 7
LA Area, mm^2^	26 ± 6
Patients presenting in AF, AF duration, months	9.6 ± 10.2
< 12 months	74 (76)
≥ 12 months	23 (24)
Patient presenting in SR, *n* (%)	48 (49)
Patients with more than 1 DCC, *n* (%)	54 (56)
Number of AADs before CA, mean ± std	2 ± 1
Patients on amiodarone before CA, *n* (%)	40 (41)

*Baseline characteristics of the AF population; std, standard deviation; DCC, electrical cardioversion; AAD, antiarrhythmic drugs*.

This study was carried out in accordance with the recommendations of the protocol CARRY, ID-RCB: 2015-A00401-48, Comité de Protection des Personnes Sud-Ouest et Outre Mer III. The protocol was approved by the Comité de Protection des Personnes Sud-Ouest et Outre Mer III. All subjects gave written informed consent in accordance with the Declaration of Helsinki.

### BSPM acquisition and preprocessing

**Figure 1:**
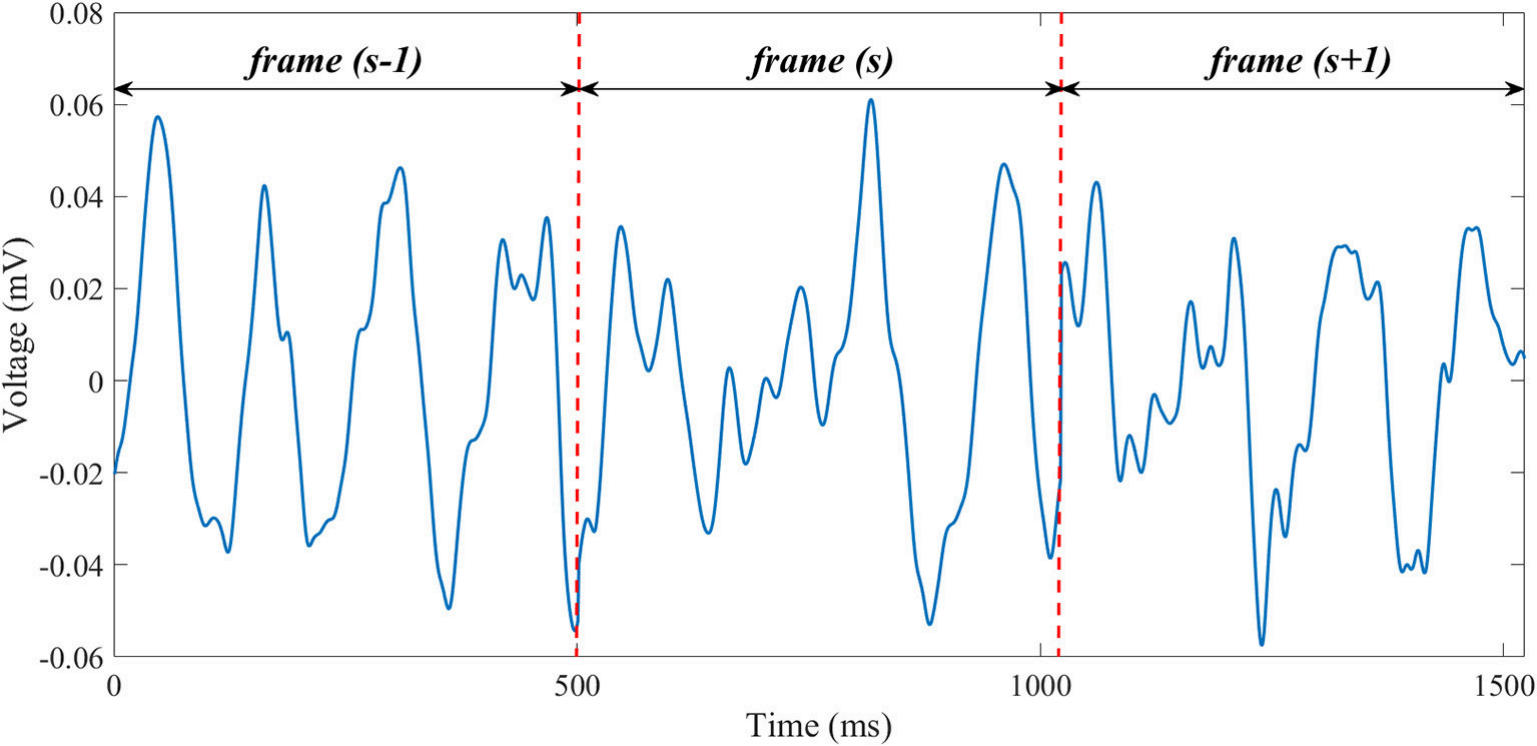
A representative example of f-wave signal extracted from a BSPM recording in an AF patient (lead 1). Concatenated, preprocessed TQ intervals are separated by dashed, red, vertical lines.

BSPMs were recorded with a 252-lead vest (CardioInsight, Medtronic, MN) in AF patients before undergoing CA at a sampling frequency of 1 kHz. Mean duration of the signals was 7 ± 4 s. TQ intervals were segmented from BSPMs with long ventricular pauses (≥ 1 s), either spontaneous or induced by diltiazem. Since the outcome of the data decomposition techniques applied in this study was not affected by the specific temporal location of each signal sample, TQ intervals could be concatenated and mean-centered to form the atrial activity signal. Baseline wandering was removed using the median estimation method (Sörnmo and Laguna, 2005). Atrial fibrillatory wave (f-wave) signals were arranged as a *L* × *N* matrix $Y=\left[y\left(1\right)\ldots \;y\left(N\right)\right]\in \;{ℜ}^{L\times \;N}$ where *L* = 252 is the number of BSPM leads, and *N* the number of samples. Electrodes with excessive noise level were discarded after signal visual inspection, thus in certain cases less than *L* electrodes were retained. A representative f-wave signal is reported in Figure 1.

### Electrophysiological atrial mapping

Intracardiac electrograms (EGMs) were continuously recorded through a computer-based digital amplifier/recorder system (Labsystem Pro, Bard Electrophysiology). Baseline CL was measured in the left atrial appendage (LAA), and monitored during the procedure to assess local CA impact. For AF electrophysiological study, we used a 20-pole steerable mapping catheter with a five-branched star design (1-mm electrodes separated by 4-mm interelectrode spacing) spanning a surface with a diameter of 3.5 cm (PentaRay, Biosense-Webster). A steerable decapolar catheter (5-mm interelectrode spacing, Xtrem, Sorin Medical, Montrouge, France) was also positioned in the coronary sinus.

### CA protocol

**Figure 2:**
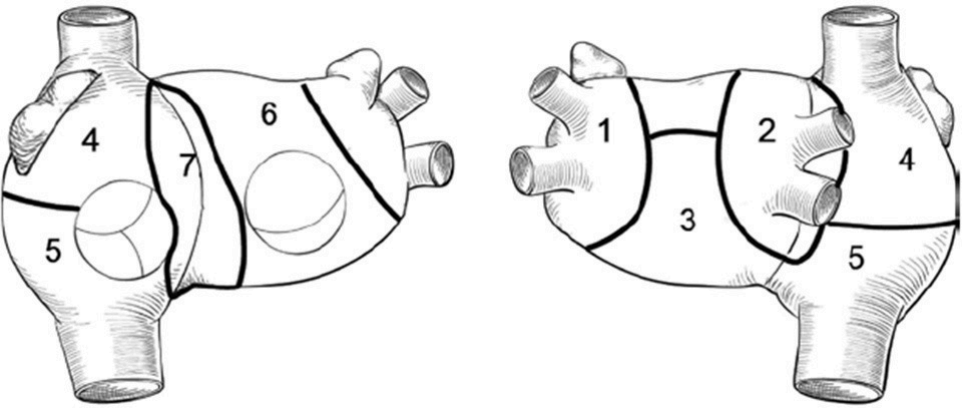
Biatrial schematic representation: 1, left pulmonary veins and LAA; 2, right pulmonary veins and posterior interatrial groove; 3, inferior and posterior left atrium; 4, upper half of right atrium and appendage; 5, lower half of right atrium; 6, anterior left atrium and roof; 7, anterior interatrial groove.

For the sake of the ablation strategy analysis, the computed tomography–reconstructed biatrial anatomy was divided into 7 regions (Figure 2).

The ablation was sequentially performed in the LA in the decreasing order of arrhythmogenic activity as estimated through noninvasive phase mapping (Haissaguerre et al., 2014) until AF terminated. Briefly, the acquisition system described in section Electrophysiological Atrial Mapping enabled the estimation of unipolar epicardial EGMs from body surface signals. Color-coded phase maps were derived from EGM phase signals by plotting the instantaneous phase values on personalized 3D biatrial geometry, which was previously reconstructed through CT scan in each patient. Surrogates of the depolarization and repolarization wavefronts were computed from the isophase values, equal to π/2 and –π/2, respectively. CA targets were identified in correspondence to phase singularity points, around which phase spanned the entire range between the two aforementioned values, as they identify AF reentrant sources (Jalife, 2003). The AF wavefront sequences detected were accumulated in a single spatiotemporal density map, displaying the distribution of active driver zones and passive conduction areas. AF drivers were classified as focal, when centrifugal activation originated from a point or an area, or reentrant, when at least 1 complete wave rotation around a center on phase progression could be tracked. Right atrium (RA) was also inspected and targeted if AF could not terminate after LA ablation. An irrigated-tip quadripolar catheter with a distal 3.5-mm tip and three 1-mm proximal electrodes with interelectrode distance of 2, 5, and 2 mm (Thermocool, Biosense-Webster) was used for AF ablation. The procedural endpoint was AF termination, i.e., conversion of AF either to SR or to intermediate atrial tachycardia (AT). AF CL was determined simultaneously in the RA with the mapping catheter and in the LAA with the ablation catheter, before and after ablation of each region, by automatically averaging 30 consecutive cycles (Bard Electrophysiology). If AF termination could not be achieved by CA, electrical cardioversion was performed.

### Theoretical basis of PCA

We investigated whether we could measure AF complexity as a function of the ability of PCA to compress the input BSPM signal into a few components while retaining the maximum amount of information as measured by variance. To this end, BSPMs were divided in *N*_*s*_ = 500-ms segments, and in each frame (*s*) singular value decomposition of the input data *Y*^(*s*)^ was performed as in Bonizzi et al. (2010); Meo et al. (2013a,b, 2017):

$$Y^{\left(s\right)}=USV^{T}$$

Where **U** and **V** represent the left and right singular vectors of **Y**^(*s*)^ respectively, and the diagonal matrix **S** contains the singular values σ_*l*_, *l* = 1, …, *L*, each associated with the principal components (PCs) **X**^(*s*)^, which are mutually uncorrelated and linked with the BSPM observations through the linear relation:

$$Y^{\left(s\right)}=M^{\left(\text{s}\right)}X^{\left(\text{s}\right)}$$

Where ${\text{M}}^{\left(s\right)}=\text{US}/\sqrt{N_{s}}$ represents the PCA transfer matrix. PCs are computed and ordered so that the first few retain most of the variance present in the input signals.

### Measuring AF spatiotemporal complexity

In line with (Bonizzi et al., 2010; Di Marco et al., 2012), we used the distance between the input BSPM signal and its rank-3 PCA approximation to measure AF organization. Since heart electrical activity on surface recordings can be well approximated by an electric dipole (Holt et al., 1969) and most of the body surface potential energy can be adequately characterized by the first 3 PCs (Lux et al., 1981), we hypothesized that organized atrial activity could be accurately retained by a 3D subspace as spanned by the first 3 columns of the PCA mixing matrix ${\text{M}}_{3}^{\left(s\right)}$. By contrast, more complex and unpredictable patterns will require a higher number of PCs to be described with sufficient accuracy, therefore the subspace chosen will yield a higher PCA reconstruction error. We introduced the nondipolar component index (NDI) to quantify the residual amount of energy which was retained by the PCA eigenvalues σ_*l*_, *l* = 4, …, *L*, outside the projection subspace spanned by the columns ${\text{M}}_{3}^{\left(s\right)}$:

$$\begin{array}{l} \text{NDI}=1-\frac{\sum_{l=1}^{3}\sigma_{l}}{\sum_{l=1}^{L}\sigma_{l}}. \end{array}$$

The global NDI parameter was determined as the average of all the values computed in each frame and served as a marker of AF complexity, with higher values denoting more disorganized and irregular signal waveforms. To increase statistical confidence, we required a minimum BSPM duration of 1 s so as to compute the NDI as the average of at least 2 values in the examined recording.

### Comparison with patient's clinical characteristics

From previous studies (Rostock et al., 2011; Scherr et al., 2015) it is known that some clinical parameters are predictive of favorable CA outcomes, such as a shorter AF duration and smaller LA size. As a consequence, the proposed signal processing methodology has been compared to the maximum continuous AF duration and LA area measured with transesophageal echocardiography.

### Comparison with other descriptors of AF complexity on surface recordings

Our multilead approach has been compared with some traditional single-lead markers of AF organization from surface recordings. To this end, NDI was also computed on a subset of BSPM electrodes at the locations of standard ECG leads and denoted NDI_ECG_, in order to verify whether this ensemble of electrodes could equally allow for a thorough characterization of AF spatiotemporal organization. As in Meo et al. (2013a), leads III and augmented leads aV_R_, aV_L_, and aV_F_ were not included as they are linearly dependent on the other frontal leads. In order to verify whether any additional information could derived from posterior BSPM leads, alternative ECG lead placement configurations were also tested. Accordingly, we assessed NDI in the optimized atrial cardiogram (OACG) system proposed in Ihara et al. (2007); van Oosterom et al. (2007), including five of the standard ECG leads (I, II, III, V_1_, and V_4_), three electrodes on the chest (V_1_S, above V_1_; V_2_RS, at the right of V_1_S; V_LC_, below the left clavicle), and a posterior one (V_1_P, at the same level as V_1_), for a total of nine electrodes. NDI computation was also performed in the extended ECG described in Petrutiu et al. (2009), consisting of 15 leads, i.e., the standard 12 ECG leads and three posterior leads V_7_, V_8_, and V_9_, which are placed below the left scapula, at the same level as V_6_, and are considered to better reflect LA activity than conventional precordial leads.

In keeping with (Nault et al., 2009; Cheng et al., 2013), f-wave amplitude A_V1_ was computed in V_1_ using a custom algorithm described in Meo et al. (2013a) and based on the interpolation of atrial signal local extrema through polynomial envelopes. In the same ECG lead AF CL was also considered as in Matsuo et al. (2009). Local maxima above a voltage threshold equal to 0.01 mV were automatically detected based on derivative sign change and a global marker CL_V1_ was obtained by averaging all CLs longer than 90 ms, so as to reject the influence of spurious local extrema.

Some multilead methods were also investigated and compared to our approach. As in Bonizzi et al. (2010), the normalized mean square error (NMSE) between the input BSPM signal and its rank-3 PCA reconstruction was determined in V_1_ and denoted *NMSE*_3_; the same tuning parameters suggested in that study were set. A multilead extension of this parameter introduced in Meo et al. (2013b) was also considered, and AF complexity was quantified as a weighted mean on NMSE values determined in multiple electrodes in the original signal and denoted WNMSE_BSPM_. Finally, a multilead characterization of f-wave amplitude as illustrated in Meo et al. (2013a) was also applied to our AF database, and a median descriptor of atrial amplitude of the rank-1 PCA estimation extracted from the input ECG was computed (A_BSPM_). All the BSPM-derived parameters of AF organization were also computed in the other ECG configuration previously described, thus yielding WNMSE_ECG_ and A_ECG_ for the 12-lead ECG counterpart of WNMSE_BSPM_ and A_BSPM_, WNMSE_OACG_ and A_OACG_ in the OACG system, and WNMSE_ECG15_ and A_ECG15_ in the extended 15-lead ECG, respectively.

### Evaluation of the clinical value of AF complexity markers

Body surface AF complexity was linked to the NDI and compared to patient's pathophysiology, meant in terms of characteristics of the underlying atrial substrate and severity of disease. Accordingly, we investigated whether rapid AF activities as measured on the baseline CL would reflect on the surface and correlate with NDI, as intracardiac AF CL is regarded as a surrogate of local refractory periods (Kim et al., 1996) and shortens with maintenance of the arrhythmia. Additionally, the relation between the proposed noninvasive index and continuous AF duration was examined. Longer AF duration was proven to be associated with a higher number of atrial AF driving sources, both focal and rotational (Lim et al., 2017), and a more complex substrate, i.e., a higher number of activation wavefronts and breakthrough waves, electrical dissociation, slower conduction and higher fractionation (De Groot et al., 2010; Lau et al., 2017). Accordingly, we hypothesized that higher NDI should be observed in long-lasting AF patients (≥12 months, 23 patients) rather than in persistent forms (<12 months, 74 patients).

Our PCA-based feature was also compared to the ablation strategy. We assumed that more severe AF forms will be more difficult to be treated, as not only the number of driving sources will be higher, but they will also appear in a higher number of sites (Haissaguerre et al., 2014; Lim et al., 2017). Therefore, we expected that CA procedures will be longer (>30 min) in terms of the amount of radiofrequency energy emission required for tissue cauterization and a higher number of atrial regions (≥4) will have to be targeted to accomplish CA successfully. Moreover, acute AF termination is considered less likely to be achieved.

The same analysis was led for the markers of AF organization reported in section Comparison With Other Descriptors of AF Complexity on Surface Recordings.

### Statistical analysis and classification performance assessment

All continuous variables were expressed as mean ± standard deviation. Parameters' distribution was checked using a Lilliefors test. For normally distributed data, intergroup differences were verified by an unpaired Student's *t*-test with Welch's correction for unequal group variances and sizes. Otherwise, a Wilcoxon's rank sum test was applied. For multivariate comparisons, one-way analysis of variance (ANOVA) was applied to normally distributed data, otherwise a Kruskall-Wallis test was used. Statistical tests were considered significant if their *p-*value was below 0.05.

We reported the area under the curve (AUC) output by the receiver operating characteristic (ROC) analysis as an index of univariate prediction performance for all AF complexity parameters. Additionally, the rates of correct detections per group were expressed in terms of the sensitivity and the specificity (i.e., the fraction of true positive and true negative cases correctly identified, respectively) associated with the optimal cutoff. Accordingly, CA procedures performed in long-lasting AF patients, with longer ablation time and a higher number of atrial targets were associated with higher AF complexity and therefore referred to as positive cases, whereas persistent AF forms and less extensive ablations (in terms of radiofrequency energy emission duration and number of regions) were regarded as negative cases.

Finally, we verified whether the evaluation of AF ablation impact (in terms of procedure outcome, duration and number of targets) based on patient's clinical data only could benefit from the integration of information about AF complexity as quantified by the aforementioned indices. Accordingly, a subset of data was used for training, whereas the other samples formed the validation set. To evaluate the ability of the multivariate features to predict ablation outcome and the number of ablated atrial sites, features from 77 patients were included in the training, whereas the remaining ones were used for validation. By contrast, since AF ablation duration had been measured only for successful procedures, smaller datasets were considered accordingly (62 training samples and 15 validation samples). Only markers of AF complexity highlighting statistically significant intergroup differences (*p-*value ≤ 0.05) were investigated. Patient's clinical data included: age, AF duration, LA area, LVEF. As in Lankveld et al. (2016b); Zeemering et al. (2017), multivariate prediction models combining clinical data (*F*_*CLIN*_) and each of the retained signal complexity parameters (*F*_*CLIN*+*SIG*_) were built by logistic regression (LR); 15-fold cross validation was performed in order to get an unbiased evaluation of a model fit on the training dataset. The output model was then applied to the validation set to determine the LR probability estimates and assign them to the related category. Prediction performance in the training and validation phase was assessed by ROC analysis as for the univariate parameters. Training and validation of classification models were first performed on multivariate features depending on patient's clinical information only (*F*_*CLIN*_). The same procedure was applied again to multivariate classifiers obtained by integrating clinical data with the parameter of signal complexity under exam (*F*_*CLIN*+*SIG*_). Classification models based on clinical data only were trained, tested and re-evaluated each time a signal complexity feature was examined, so as to specifically investigate the effect of the presence/absence of each measure of AF complexity and ensure a consistent comparison between classification scores always on the same datasets, especially in case of missing data. The predictive accuracy of AF duration was tested using the same methodology as well and compared with the clinical set of variables $F_{CLIN^{*}}$ (including patient's age, LA area and LVEF). The improvement in classification accuracy provided by the integration of the signal-derived parameter was evaluated in terms of the net reclassification index (NRI), which is defined as the sum of the net percentages of correctly reclassified samples in the categories of interest (Pencina et al., 2008). Null NRI values denote the absence of improvement in the classification by adding a new variable. The null hypothesis NRI = 0 was verified by a z-test and considered statistically significant if *p-*value was <0.05.

## Results

### Electrophysiological mapping and ablation

Baseline AF CL was 178 ± 55 ms. It was shorter than 160 ms in 33 patients, between 160 and 180 ms in 15 patients, and longer than 180 ms in the remaining ones. LA area was 26 ± 6 cm^2^. Out of 97 patients, 17 of them underwent a redo ablation (17%). AF induction was performed in 48 patients (49%) prior to CA. AF converted to SR in 27 patients, to AT in 50 patients (global AF termination rate: 79%). Intracardiac AF CL was 177 ± 40 ms in AF-free patients, whereas it was 157 ± 26 ms for failed procedures (*p* = 0.0586). Mean ablation duration was 35 ± 21 min (<30 min in 38 out of 97 patients) and 4 ± 2 regions (between 1 and 3 sites in 36 AF patients) were targeted by CA. BSPM recordings acquired in 3 subjects were discarded from our analysis as their duration was below our requirements.

### Assessment of AF complexity in body surface potentials

**Figure 3:**
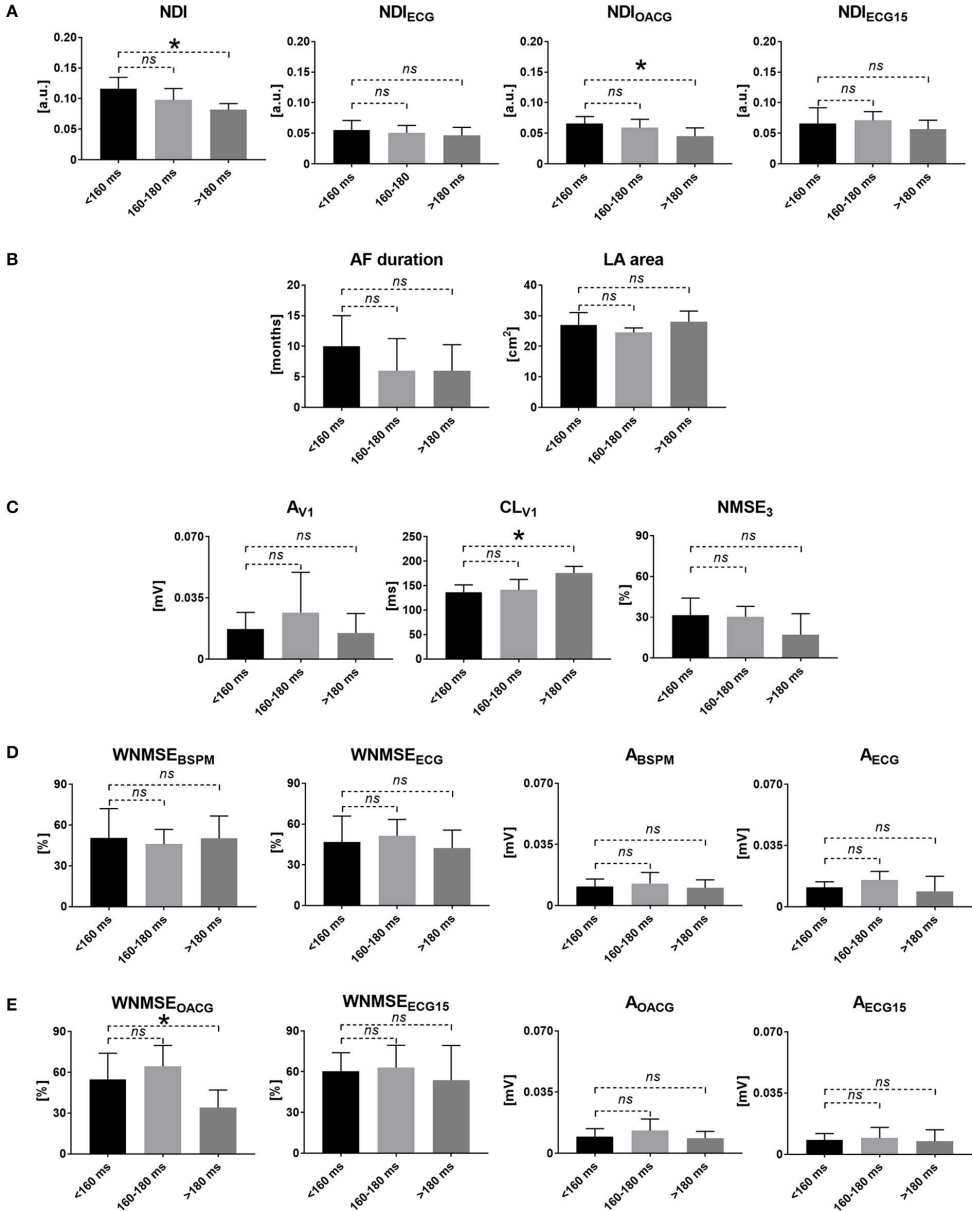
Noninvasive markers of AF complexity and AF CL: correlation of surface signal features with AF disease chronification based on atrial AF CL. **(A)** The NDI index measured in BSPMs (NDI, left) and the standard ECG subset (NDI_ECG_, right). **(B)** Clinical measures of cardiac activity: AF duration (left) and LA area (right). **(C)** Single-lead descriptors of atrial amplitude (A_V1_, left), surface CL (CL_V1_, middle) and PCA estimation error (NMSE_3_, right) in V1. **(D)** Multilead PCA reconstruction error (WNMSE_BSPM_, left) and f-wave amplitude (A_BSPM_, right) computed in BSPMs and in the standard ECG subset (WNMSE_ECG_ and A_ECG_). **(E)** Multilead PCA reconstruction error (WNMSE_OACG_, left) and f-wave amplitude (A_OACG_, right) computed in the modified OACG system and in the extended 15-lead ECG (WNMSE_ECG15_ and A_ECG15_). ^*^p < 0.05 vs. <160 ms; *ns*, not significant; a.u., arbitrary units.

Results related to the analysis of the relation between the BSPM indices of AF organization and intracardiac AF CL are shown in Figure 3.

A significantly inverse correlation between the NDI and the CL measured in the LAA was demonstrated, with high complexity values observed in very advanced AF forms (AF CL <160 ms) and progressively decreasing in less severe cases. Similar results could be retrieved in the alternative OACG lead configuration. A significantly direct correlation with intracardiac CL was remarked for CL_V1_ instead, i.e., higher body surface complexity as quantified by high NDI values reflected faster activations of the atrial substrate. Higher values of the multilead index of AF complexity WNMSE_OACG_ were also associated with more rapid intracardiac AF CL.

**Figure 4:**
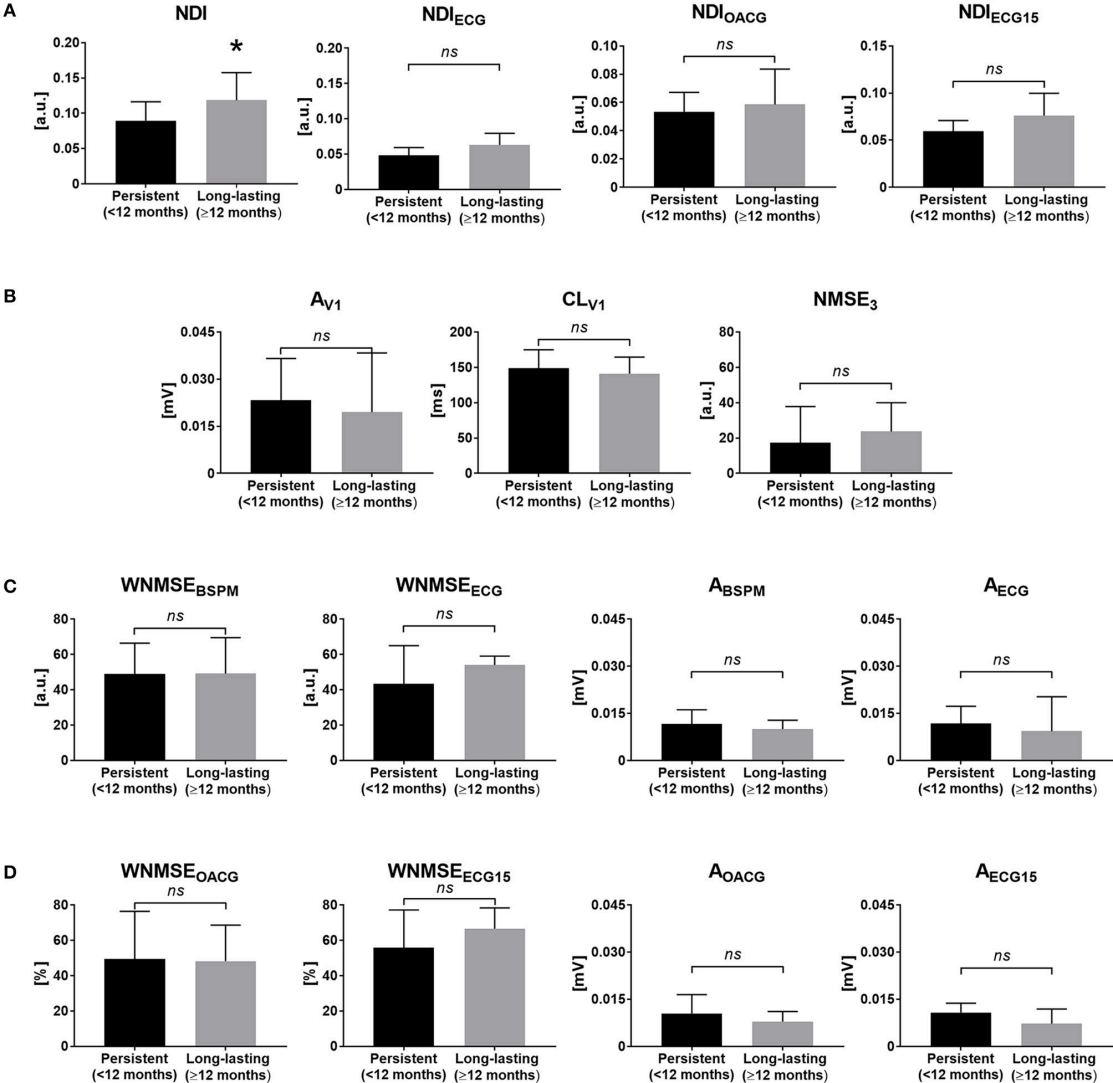
Noninvasive markers of AF complexity and continuous AF duration: correlation of surface signal features with AF disease chronification based on the duration of the last continuous episode. **(A)** The NDI index measured in BSPMs (NDI, left) and the standard ECG subset (NDI_ECG_, right). **(B)** Single-lead descriptors of atrial amplitude (A_V1_, left), surface CL (CL_V1_, middle) and PCA estimation error (NMSE_3_, right) in V1. **(C)** Multilead PCA reconstruction error (WNMSE_BSPM_, left) and f-wave amplitude (A_BSPM_, right) computed in BSPMs and in the standard ECG subset (WNMSE_ECG_ and A_ECG_). **(D)** Multilead PCA reconstruction error (WNMSE_OACG_, left) and f-wave amplitude (A_OACG_, right) computed in the modified OACG system and in the extended 15-lead ECG (WNMSE_ECG15_ and A_ECG15_). ^*^p < 0.05 vs. persistent AF; *ns*, not significant; a.u., arbitrary units.

Statistical analysis outcome for the signal features assessed in persistent and long-lasting AF cases is shown in Figure 4.

LA surface did not highlight significant differences between the two groups of patients (persistent AF: 27 ± 6 cm^2^; long-lasting AF: 25 ± 7 cm^2^, *p* = 0.38). NDI computed from BSPMs was the only one parameter highlighting significantly higher signal disorganization in more advanced AF forms, which were more accurately discriminated by the parameter according to the ROC analysis, as shown in Table 2.

**Table 2:** ROC analysis of the AF complexity features and AF duration.

	**AUC [%]**	**Sensitivity [%]**	**Specificity [%]**
NDI [a.u]	**70**	**55**	**79**
LA area [cm^2^]	59	36	79
A_V1_ [mV]	52	55	60
CL_V1_ [ms]	60	45	82
NMSE_3_ [%]	51	55	60
WNMSE_BSPM_ [%]	58	86	42
A_BSPM_ [mV]	59	91	30
NDI_ECG_ [a.u]	63	58	84
WNMSE_ECG_ [%]	58	55	68
A_ECG_ [mV]	54	58	64
NDI_OACG_ [a.u]	59	37	90
WNMSE_OACG_ [%]	57	47	69
A_OACG_ [mV]	58	79	48
NDI_ECG15_ [a.u]	65	63	76
WNMSE_ECG15_ [%]	56	95	24
A_ECG15_ [mV]	61	42	85

*Assessment of the ability of the AF organization markers to discriminate between persistent and long-lasting AF patients. Sensitivity and specificity indicate the rate of correct detections in the long-lasting and persistent AF patients' groups, respectively. Results for the parameters with the highest classification performance (AUC≥70%) are highlighted in boldface. AUC, area under curve; a.u., arbitrary units*.

The impact of AF complexity on the procedural time was quantified in Table 3.

**Table 3:** Noninvasive markers of AF complexity and ablation time.

	**Ablation time (≤ 30 min)**	**Ablation time (> 30 min)**	***p*-value**
NDI [a.u]	0.086 ± 0.036	0.107 ± 0.032	**0.0009**
AF duration [months]	5.9 ± 6.0	9.2 ± 8.7	**0.030**
LA area [cm^2^]	26 ± 6	26 ± 7	0.65
A_V1_ [mV]	0.022 ± 0.015	0.032 ± 0.023	0.063
CL_V1_ [ms]	168 ± 34	145 ± 32	**0.002**
NMSE_3_ [%]	23.4 ± 17.7	25.2 ± 19.4	0.71
WNMSE_BSPM_ [%]	42.4 ± 26.0	53.8 ± 26.3	0.064
A_BSPM_ [mV]	0.013 ± 0.006	0.012 ± 0.005	0.81
NDI_ECG_ [a.u]	0.047 ± 0.016	0.055 ± 0.023	0.26
WNMSE_ECG_ [%]	42.4 ± 25.3	46.8 ± 24.0	0.49
A_ECG_ [mV]	0.010 ± 0.006	0.015 ± 0.008	**0.017**
NDI_OACG_ [a.u]	0.051 ± 0.022	0.060 ± 0.025	0.15
WNMSE_OACG_ [%]	49.6 ± 27.6	48.0 ± 25.5	0.72
A_OACG_ [mV]	0.010 ± 0.0006	0.013 ± 0.007	0.11
NDI_ECG15_ [a.u]	0.058 ± 0.019	0.071 ± 0.029	**0.043**
WNMSE_ECG15_ [%]	55.6 ± 25.0	55.3 ± 26.6	0.80
A_ECG15_ [mV]	0.009 ± 0.004	0.013 ± 0.007	**0.025**

*Correlation of body surface signal features with the duration of radiofrequency emission duration to achieve procedural AF termination; p values in boldface are statistically significant; a.u., arbitrary units*.

Significantly low NDI values characterized shorter CA procedures, whereas higher complexity was measured by the index in longer ablations. Similarly, NDI_ECG15_ put more disorganized signal waveforms from the modified 15-lead ECG in relation to longer procedural time. Finally, the multilead assessment of f-wave amplitude in the same ECG lead system underlined statistically significant differences between the groups examined, even if it unexpectedly correlated lower amplitude values with shorter CA. Among all multilead descriptors of AF complexity, NDI assessed in BSPMs was the only one exhibiting a high predictive power as well, as confirmed by the ROC analysis in Table 4.

**Table 4:** ROC analysis of the AF complexity features and ablation time.

	**AUC [%]**	**Sensitivity [%]**	**Specificity [%]**
NDI [a.u]	**72**	**71**	**78**
AF duration [months]	64	28	97
LA area [cm^2^]	53	51	66
A_V1_ [mV]	64	61	73
CL_V1_ [ms]	**73**	**74**	**70**
NMSE_3_ [%]	53	35	83
WNMSE_BSPM_ [%]	63	79	47
A_BSPM_ [mV]	52	44	71
NDI_ECG_ [a.u]	59	63	61
WNMSE_ECG_ [%]	56	48	70
A_ECG_ [mV]	68	57	79
NDI_OACG_ [a.u]	61	43	82
WNMSE_OACG_ [%]	53	90	29
A_OACG_ [mV]	61	37	89
NDI_ECG15_ [a.u]	65	70	61
WNMSE_ECG15_ [%]	52	23	93
A_ECG15_ [mV]	67	47	86

*Assessment of the ability of the AF organization descriptors to distinguish between short and long AF ablation procedures. Sensitivity and specificity indicate the percentage of interventions correctly identified by the signal features based on the duration of radiofrequency emission duration to AF termination, i.e., longer/shorter than 30 min, respectively. Results for the parameters with the highest classification performance (AUC≥70%) are highlighted in boldface. AUC, area under curve; a.u., arbitrary units*.

By contrast, NDI_ECG15_ was characterized by low predictive accuracy as proven by the ROC analysis. Long-lasting AF patients also underwent significantly longer CA procedures, although the predictive value of AF duration was quite low. Surface CL CL_V1_ was also significantly shorter in patients requiring longer CA procedures, and ROC analysis yielded predictive results as well. Unexpectedly, the multilead marker of f-wave amplitude A_ECG_ was significantly higher in patients undergoing longer CA procedures. The same results were output by A_ECG15_ in the extended 15-lead ECG set. However, in both cases the ROC analysis underlined low predictive performance, due to the inability to correctly identify long CA procedures based on AF complexity content.

In Table 5 we illustrated the relation between AF complexity and CA effectiveness, expressed in terms of the number of atrial regions to be ablated to achieve the procedural endpoint.

**Table 5:** Noninvasive markers of AF complexity and number of CA targets.

	**Number of CA targets (<4)**	**Number of CA targets (≥4)**	***p*-value**
NDI [a.u]	0.086 ± 0.030	0.111 ± 0.037	**0.002**
AF duration [months]	4.6 ± 3.4	12.6 ± 13.1	**<0.0001**
LA area [cm^2^]	27 ± 6	26 ± 6	0.56
A_V1_ [mV]	0.024 ± 0.016	0.030 ± 0.024	0.27
CL_V1_ [ms]	167 ± 35	147 ± 29	**0.007**
NMSE_3_ [%]	21.8 ± 18.0	24.4 ± 18.5	0.54
WNMSE_BSPM_ [%]	43.3 ± 27.6	52.8 ± 24.7	0.065
A_BSPM_ [mV]	0.012 ± 0.006	0.011 ± 0.005	0.32
NDI_ECG_ [a.u]	0.048 ± 0.019	0.055 ± 0.022	0.18
WNMSE_ECG_ [%]	39.1 ± 24.9	46, 59 ± 23.4	0.49
A_ECG_ [mV]	0.011 ± 0.006	0.013 ± 0.008	0.26
NDI_OACG_ [a.u]	0.054 ± 0.027	0.059 ± 0.022	0.18
WNMSE_OACG_ [%]	46.9 ± 29.1	50.8 ± 24.1	0.62
A_OACG_ [mV]	0.012 ± 0.006	0.0012 ± 0.007	0.71
NDI_ECG15_ [a.u]	0.059 ± 0.021	0.071 ± 0.029	0.11
WNMSE_ECG15_ [%]	51.7 ± 26.2	57.4 ± 22.9	0.75
A_ECG15_ [mV]	0.010 ± 0.005	0.011 ± 0.006	0.63

*Correlation of BSPM features with the number of atrial sites ablated to achieve procedural AF termination; p values in boldface are statistically significant; a.u., arbitrary units*.

Also in this case, CA procedures requiring a lower number of targets to terminate AF characterized more organized waveforms, quantified by significantly lower NDI and higher CL_V1_ values. Nevertheless, both indices were characterized by low predictive accuracy, as confirmed by the ROC analysis in Table 6. Longer AF duration was also predictive of a more extensive ablation.

**Table 6:** ROC analysis of the AF complexity features and number of CA targets.

	**AUC [%]**	**Sensitivity [%]**	**Specificity [%]**
NDI [a.u]	69	67	71
AF duration [months]	**75**	**64**	**75**
LA area [cm^2^]	54	67	45
A_V1_ [mV]	58	54	64
CL_V1_ [ms]	68	85	39
NMSE_3_ [%]	54	35	82
WNMSE_BSPM_ [%]	61	88	38
A_BSPM_ [mV]	56	23	92
NDI_ECG_ [a.u]	59	37	92
WNMSE_ECG_ [%]	59	69	50
A_ECG_ [mV]	58	80	38
NDI_OACG_ [a.u]	59	47	77
WNMSE_OACG_ [%]	53	82	35
A_OACG_ [mV]	53	86	31
NDI_ECG15_ [a.u]	61	37	88
WNMSE_ECG15_ [%]	56	74	42
A_ECG15_ [mV]	53	92	19

*Assessment of the ability of the AF organization descriptors to identify extensive ablation procedures in terms of the number of atrial sites targeted to achieve AF termination. Sensitivity and specificity indicate the percentage of procedures correctly classified by the signal features based on the number of ablation targets, i.e., more/less than 4 sites, respectively. Results for the parameters with the highest classification performance (AUC≥70%) are highlighted in boldface. AUC, area under curve; a.u., arbitrary units*.

**Figure 5:**
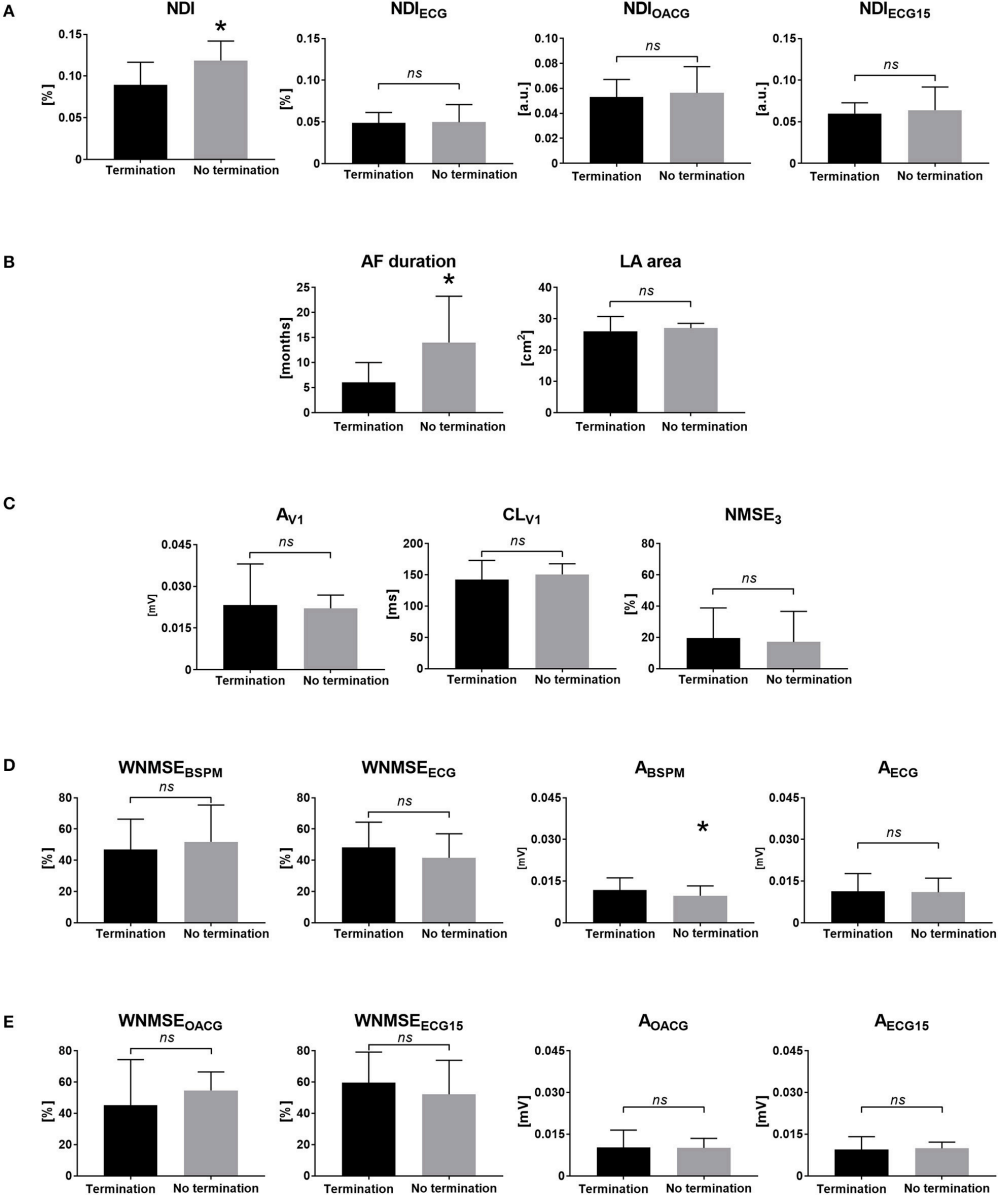
Noninvasive markers of AF complexity and CA outcome: correlation of BSPM features with the acute CA outcome. **(A)** The NDI index measured in BSPMs (NDI, left) and the standard ECG subset (NDI_ECG_, right). **(B)** Clinical measures of cardiac activity: AF duration (left) and LA area (right). **(C)** Single-lead descriptors of atrial amplitude (A_V1_, left), surface CL (CL_V1_, middle) and PCA estimation error (NMSE_3_, right) in V1. **(D)** Multilead PCA reconstruction error (WNMSE_BSPM_, left) and f-wave amplitude (A_BSPM_, right) computed in BSPMs and in the standard ECG subset (WNMSE_ECG_ and A_ECG_). **(E)** Multilead PCA reconstruction error (WNMSE_OACG_, left) and f-wave amplitude (A_OACG_, right) computed in the modified OACG system and in the extended 15-lead ECG (WNMSE_ECG15_ and A_ECG15_). ^*^p < 0.05 vs. AF termination; *ns*, not significant; a.u., arbitrary units.

Finally, in Figure 5 the ability of the BSPM indices to assess short-term CA outcome was examined. Low NDI values were predictive of procedural AF termination, whereas AF forms which were less likely to be successfully converted to other rhythms by CA presented higher disorganization, quantified by higher NDI.

CA outcome prediction performance was assessed by ROC analysis in Table 7.

**Table 7:** ROC analysis of the AF complexity features and CA outcome.

	**AUC [%]**	**Sensitivity [%]**	**Specificity [%]**
NDI [a.u]	69	70	67
AF duration [months]	**70**	**55**	**87**
LA area [cm^2^]	51	65	53
A_V1_ [mV]	51	84	33
CL_V1_ [ms]	52	37	75
NMSE_3_ [%]	56	32	85
WNMSE_BSPM_ [%]	57	95	26
A_BSPM_ [mV]	67	85	40
NDI_ECG_ [a.u]	56	37	81
WNMSE_ECG_ [%]	53	63	49
A_ECG_ [mV]	51	84	33
NDI_OACG_ [a.u]	57	37	88
WNMSE_OACG_ [%]	52	63	52
A_OACG_ [mV]	50	98	24
NDI_ECG15_ [a.u]	58	47	85
WNMSE_ECG15_ [%]	51	95	26
A_ECG15_ [mV]	54	84	33

*Assessment of the ability of the AF organization descriptors to predict AF termination by CA. Sensitivity and specificity indicate the rate of detection of successful and failed ablation procedures, respectively. Results for the parameters with the highest classification performance (AUC≥70%) are highlighted in boldface. AUC, area under curve; a.u., arbitrary units*.

It yielded AUC = 69%, sensitivity = 70%, specificity = 67% for NDI. Moreover, patients with longer AF episodes were significantly less likely to experience procedural success. AF termination by CA was also predicted by significantly higher f-wave amplitude, quantified by higher A_BSPM_ values, but with a poor ROC analysis outcome. The multilead BSPM descriptors of AF organization were also computed in the modified configurations of the standard 12-lead ECG, and the statistical analysis results were shown together with the aforementioned parameters. Nevertheless, overall those parameters did not yield significant results (*p* > 0.05).

We also evaluated the classification accuracy of each of the examined AF complexity markers in combination with AF patients' clinical information.

In Table 8 the ability of multivariate classifiers to distinguish between short and long CA procedures was reported.

**Table 8:** Multivariate classification performance based on ablation duration and NRI assessment.

	***F***_***CLIN***_						***F***_***CLIN***+***SIG***_							
	**Training**			**Validation**			**Training**			**Validation**				
	**AUC [%]**	**Sensitivity [%]**	**Specificity [%]**	**AUC [%]**	**Sensitivity [%]**	**Specificity [%]**	**AUC [%]**	**Sensitivity [%]**	**Specificity [%]**	**AUC [%]**	**Sensitivity [%]**	**Specificity [%]**	**NRI [a. u.]**	***p*-value**
NDI [a.u.]	56	42	65	29	100	14	62	45	**65**	65	80	57	−0.04	0.89
AF duration [months]	41	32	52	49	40	86	53	39	68	29	100	14	0.18	0.56
CL _V1_ [ms]	59	42	71	29	100	14	61	42	71	**85**	100	60	0.12	0.32
A_BSPM_ [mV]	54	35	65	29	100	14	54	35	65	**85**	100	80	0.23	0.37
NDI_ECG15_ [a.u]	57	19	66	43	100	29	63	46	55	**85**	100	60	−0.16	0.50
A_ECG15_ [mV]	57	35	71	29	100	14	61	39	71	**70**	100	60	−0.04	0.89

ROC analysis of the multivariate classifiers obtained by using clinical data only (F_CLIN_) and by integrating an AF complexity descriptor (F_CLIN+SIG_) in the training and validation sets based on procedural duration. Training and validation of classification models were first performed on multivariate features depending on patient's clinical information only (F_CLIN_). The same procedure was applied again to multivariate classifiers obtained by integrating clinical data with the parameter of signal complexity under exam (F_CLIN+SIG_). Classification models based on F_CLIN_ were trained, tested and re-evaluated each time a signal complexity feature was examined. The variable F_CLIN+SIG_ including AF duration was compared to F_CLIN_^*^. Differences in classification accuracy were quantified by the NRI. Results for the parameters with the highest classification performance (AUC≥70%) are highlighted in boldface; AUC, area under curve; a.u., arbitrary units.

Clinical indices alone could not effectively discriminate ablation interventions based on their duration. Adding an AF complexity marker considerably improved the classification accuracy in the validation set in terms of AUC more clearly than in the training set for the univariate AF CL CL_V1_ and the multivariate descriptors of amplitude assessed in the full set of BSPM electrodes (A_BSPM_) and in the 15-lead ECG subset (A_ECG15_). Similar findings were made for the NDI assessed in the same lead configuration (NDI_ECG15_), but these results could not be reproduced on the original BSPM lead configuration. However, the degree of improvement of classification performance as assessed by the NRI was not statistically significant for any of these multivariate features.

In Table 9 the ability of the multivariate classifiers to characterize more extensive ablation interventions in terms of the number of procedural targets was investigated.

**Table 9:** Multivariate classification performance based on the number of ablation targets and NRI assessment.

	***F***_***CLIN***_						***F***_***CLIN***+***SIG***_							
	**Training**			**Validation**			**Training**			**Validation**				
	**AUC [%]**	**Sensitivity [%]**	**Specificity [%]**	**AUC [%]**	**Sensitivity [%]**	**Specificity [%]**	**AUC [%]**	**Sensitivity [%]**	**Specificity [%]**	**AUC [%]**	**Sensitivity [%]**	**Specificity [%]**	**NRI [a. u.]**	***p*-value**
NDI [a.u.]	**72**	79	53	64	50	86	**79**	47	79	**86**	86	83	0.24	0.28
AF duration [months]	53	96	7	40	100	17	**75**	82	52	52	71	50	0.17	0.62
CL_V1_ [ms]	**74**	80	52	52	71	50	**77**	86	41	**71**	57	100	−0.04	0.81

ROC analysis of the multivariate classifiers obtained by using clinical data only (F_CLIN_) and by integrating an AF complexity descriptor (F_CLIN+SIG_) in the training and validation sets based on the number of atrial sites targeted by ablation. Training and validation of classification models were first performed on multivariate features depending on patient's clinical information only (F_CLIN_). The same procedure was applied again to multivariate classifiers obtained by integrating clinical data with the parameter of signal complexity under exam (F_CLIN+SIG_). Classification models based on F_CLIN_ were trained, tested and re-evaluated each time a signal complexity feature was examined. The variable F_CLIN+SIG_ including AF duration was compared to $F_{CLIN}^{*}$. Differences in classification accuracy were quantified by the NRI. Results for the parameters with the highest classification performance (AUC≥70%) are highlighted in boldface; AUC, area under curve; a.u., arbitrary units.

Classification accuracy based on clinical data was improved by the introduction of the NDI marker in the validation set (AUC = 86%, sensitivity = 86%, specificity = 83%). Similarly, CA procedures could be better discriminated based on the number of ablated atrial regions by adding CL_V1_. By contrast, no benefits were provided by information about AF duration. Overall, none of these changes was statistically significant according to NRI analysis.

Finally, in Table 10 the classification performance of multidimensional predictors of CA outcome was shown.

**Table 10:** Multivariate ablation outcome classification performance and NRI assessment.

	***F***_***CLIN***_						***F***_*****CLIN***+***SIG*****_							
	**Training**			**Validation**			**Training**			**Validation**				
	**AUC [%]**	**Sensitivity [%]**	**Specificity [%]**	**AUC [%]**	**Sensitivity [%]**	**Specificity [%]**	**AUC [%]**	**Sensitivity [%]**	**Specificity [%]**	**AUC [%]**	**Sensitivity [%]**	**Specificity [%]**	**NRI [a. u.]**	***p*-value**
NDI [a.u.]	58	19	97	67	67	90	60	25	92	**70**	100	50	0	N.A
AF duration [months]	19	0	100	43	33	100	56	98	19	67	67	90	0	N.A
A_BSPM_ [mV]	59	15	97	67	67	90	66	15	97	**79**	67	100	0.25	0.32

ROC analysis of the multivariate predictors of ablation outcome obtained by using clinical data only (F_CLIN_) and by integrating an AF complexity descriptor (F_CLIN+SIG_) in the training and validation sets. Training and validation of classification models were first performed on multivariate features depending on patient's clinical information only (F_CLIN_). The same procedure was applied again to multivariate classifiers obtained by integrating clinical data with the parameter of signal complexity under exam (F_CLIN+SIG_). Classification models based on F_CLIN_ were trained, tested and re-evaluated each time a signal complexity feature was examined. The variable F_CLIN+SIG_ including AF duration was compared to F_CLIN_^*^. Differences in classification accuracy were quantified by the NRI. Results for the parameters with the highest classification performance (AUC≥70%) are highlighted in boldface; AUC, area under curve; a.u., arbitrary units; N.A., not applicable.

As in the previous case, prediction accuracy in the validation phase was higher when NDI was also included into the classification model (AUC = 70%, sensitivity = 100%, specificity = 50%). Similar remarks could be made for the single-lead CL CL_V1_. Information provided by AF duration to the classification model was poor instead, both in the training and the validation phase. However, also in this case changes in the classification scores as measured by the NRI were not statistically significant.

## Discussion

In this study we proposed a noninvasive PCA-based approach to evaluate AF complexity in BSPMs, which can be accurately characterized even in very short recordings (<10 s in our database). The algorithm overcomes limitations of QRST cancelation, which may be affected by R peak misalignment or sudden changes in signal voltage, thus minimizing the influence of residual ventricular far field. Indeed, since PCA assumptions rely on signal second-order statistics at zero time lag, i.e., the coherence between consecutive samples is neglected, the use of temporally consecutive samples is not necessary (Bonizzi et al., 2010). Furthermore, it does not require any a priori selection of specific electrodes, as it automatically condenses the most relevant signal information into a few components based on its energy content. In addition, apart from the duration of the signal to be processed, no further tuning parameters need to be set, thus making this tool easier to be implemented and integrated to AF complexity analysis than other indices from the state of the art, e.g., sample entropy (Alcaraz et al., 2011). Our methodology provided relevant insights into the characteristics of AF disease and substrate and correlated with CA strategy.

### Surface AF complexity and characteristics of AF disease and substrate

The proposed methodology can quantify AF organization in surface recordings and correlate it to the underlying electrophysiological substrate.

A decreasing trend of NDI as a function of AF CL was observed, and more rapid local activities in the atria reflected on higher complexity on body surface.

Similar evidence was found for CL_V1_, thus proving a direct correlation between the invasive measure of the atrial fibrillatory rate and the surface electrical activity, which was previously demonstrated in (Matsuo et al., 2009) as well.

By contrast, no significant correlation between continuous AF duration and body surface complexity. Indeed, this clinical parameter may not reliably reflect the properties of the underlying atrial substrate, as it is often difficult to determine, unless continuous long-term ECG monitoring is performed (Ciconte et al., 2017). However, this approach would not be helpful in asymptomatic AF patients (Ahmad and Kirchhof, 2013), whose diagnosis is still challenging. Paradoxically, current guidelines increasingly tend not to distinguish between the prognostic implications of paroxysmal vs. long-standing AF (Calkins et al., 2012). Some studies demonstrated that patients with similar clinical characteristics (including AF history) may present very different substrates (Kottkamp, 2013) and even some paroxysmal AF forms may be due to sources other than those in the pulmonary veins (Sanchez-Quintana et al., 2012). Nevertheless, the relation between number of AF driving sources and disease duration has been shown elsewhere (Lim et al., 2017), thus making it harder to reach a consensus about the role of AF duration as a marker of complexity.

Higher complexity was measured by higher NDI in patients with longer AF episode duration, which may result from a longer electrical remodeling of the atrial substrate, due to disease progression (Lau et al., 2017) and the onset of multiple sources located even outside the pulmonary vein areas (Lim et al., 2017). The strength of this correlation was also supported by the ROC analysis, confirming the ability of the proposed index to assess atrial activity rate based on body surface signal complexity.

Surprisingly, longer AF duration and larger LA area did not show any evident correlation with faster AF CLs, in contrast with evidence reported in Ammar et al. (2014). However, the same study claims that variations in intracardiac CL depend both on other patient's clinical characteristics, including age and other comorbidities, and external factors, such as pharmacological interventions, thus this finding should be investigated in a broader context.

Body surface measures of f-wave amplitude could not significantly reflect the properties of the AF wavefront propagation in terms of CL. Moreover, all amplitude features did not exhibit any significant correlation with AF duration. While some studies have discovered a correlation between atrial amplitude and AF duration and echocardiographic characteristics (Yamamoto et al., 2005), in Nault et al. (2009) and other more recent studies that finding was impossible to reproduce, thus confirming the divergence between results reported in literature.

Indices of complexity based on PCA reconstruction error (i.e., NMSE_3_ and WNMSE_BSPM_) could not effectively quantify the degree of AF chronification. In contrast with our intuition, higher PCA projection errors did not significantly reflect faster intracardiac AF activation. Additionally, the accuracy of PCA estimation was not significantly lower in long-lasting AF patients. This may be partially explained by the use of the setting proposed in the related reference studies, which may be not suitable for our signal database. Furthermore, those parameters rather aimed to quantify the degree of stationarity and repetitiveness of atrial components across the electrodes, which may not be sufficiently evident in short recordings as those examined in our study.

### Surface AF complexity and CA strategy

Our PCA-derived parameter could also quantify the impact of AF complexity on the CA therapy strategy. Indeed, higher complexity was underlined by NDI in surface recordings in patients undergoing shorter ablation procedures, and the univariate ROC analysis corroborated the ability of the index to accurately distinguish between interventions of different duration based on the signal complexity information. Furthermore, a lower number of atrial targets and higher procedural success probability were associated with more complex AF waveforms, despite the weaker predictive performance. By contrast, more disorganized AF forms were less likely to be successfully converted to other rhythms by CA, and they generally required longer interventions and a more extensive cauterization of atrial tissue.

Continuous AF duration proved to be a significant univariate predictor of CA outcome, and long-lasting cases overall required a more extensive ablation, in line with previous research (Scherr et al., 2009; Rostock et al., 2011). Despite this performance, it is essential to keep in mind some of the aforementioned limitations of AF duration, e.g., the potentially inaccurate evaluation of its value in certain patients, or the lack of correlation with the atrial substrate, which may lead to an erroneous evaluation of the ablation strategy, thus corroborating the added descriptive value from cardiac signal processing parameters. Conversely, LA surface did not show any relevant correlation with CA strategy and effectiveness. This finding may appear in contrast with current literature (Zhuang et al., 2012; Scherr et al., 2015). Nevertheless, as pointed out in Hoit (2014), despite current recommendations for LA size assessment, clinical studies report a wide variety of 1-dimensional linear and 2D area measurements, which may lead to contrasting results and a make it harder to understand the clinical value of this parameter.

Preprocedural CL measured in lead V_1_ (CL_V1_) also appeared longer in ablations characterized by a lower number of candidate atrial sites for CA and with lower amount of radiofrequency energy emission. In keeping with (Matsuo et al., 2009), this finding suggests that CA results are not caused by operator bias, but by an increased complexity of AF substrate, and it is corroborated by ROC analysis as well. However, the index was not predictive of acute AF termination by CA, in contrast with results presented in Matsuo et al. (2009).

Acute CA outcome was significantly predicted by the multilead amplitude feature A_BSPM_, which is consistent with results described in Meo et al. (2013a), despite the weak predictive performance. By contrast, its single-lead counterpart A_V1_ did not significantly discriminate between successful and failing CA procedures, which is in contradiction with evidence shown in Nault et al. (2009). However, as pointed out in the same study, f-wave amplitude measure is highly dependent on ECG acquisition modalities and it is sensitive to external artifacts, thus results reported by clinical studies are quite disparate and difficult to interpret.

Multilead PCA-based descriptors of AF organization NMSE_3_ and WNMSE_BSPM_ could not significantly quantify the impact of atrial substrate complexity on AF ablation characteristics. As explained in section Surface AF Complexity and Characteristics of AF Disease and Substrate, a potential explanation of their weak predictive performance can be the impossibility to assess the spatiotemporal variability of the atrial signal pattern in very short signals. Nevertheless, this remark should be verified by additional experiments.

### Benefits from the spatial variability of multilead recordings

All the multilead PCA-based descriptors of AF complexity obtained in BSPMs were also computed in an equivalent set of electrodes of the standard 12-lead ECG, thus yielding NDI_ECG_, WNMSE_ECG_ and A_ECG_, and the same statistical analysis was performed. Similarly, alternative body surface lead configurations were tested, i.e., the OACG system developed in Ihara et al. (2007); van Oosterom et al. (2007) and the extended 15-lead ECG system examined in Petrutiu et al. (2009), with the AF complexity markers denoted as NDI_OACG_, WNMSE_OACG_ and A_OACG_ and NDI_ECG15_, WNMSE_ECG15_, A_ECG15_, respectively.

No index from 12-led ECG did significantly correlate neither with atrial substrate properties during AF nor with CA procedure characteristics and outcome. This may be due to the inability of standard ECG to sufficiently capture the spatial variability of AF pattern wavefront, which can be instead more accurately characterized in larger sets of electrodes. Indeed, slight improvements in this characterization were observed in the OACG system, thus confirming the benefits of the analysis of the cardiac electrical activity in additional leads.

The relation between body surface complexity as measured by WNMSE_OACG_ and intracardiac AF CL in the OACG subset was also significant, but not strictly decreasing as for the aforementioned measures of AF organization, thus further investigation should be performed in the assessment of this quantitative relation. These results underlined the added value of the posterior OACG lead, which is assumed to better characterize LA activity, due to its proximity to lead M of Frank's vector lead system (Ihara et al., 2007). However, a more strategic and effective ECG lead placement configuration should be investigated in more detail in order to increase the predictive power of the related AF organization measures.

BSPM capability of providing a more comprehensive view of surface cardiac electrical activity has been previously demonstrated for ventricular electrical disorders (Robinson et al., 2009), and advances in diagnosis and therapy of supraventricular arrhythmias have been obtained as well, thanks to the more reliable identification of arrhythmogenic sources driving and sustaining the pathological rhythm (SippensGroenewegen et al., 2004; Haissaguerre et al., 2013; Yamashita et al., 2015), thus corroborating the clinical value of BSPM analysis.

Concerning the CA strategy, the NDI determined from the extended 15-lead ECG proposed in Petrutiu et al. (2009) correlated higher body surface complexity with prolonged ablation procedures, thus demonstrating that relevant insights into AF therapy can be better provided by additional leads reflecting the underlying LA activity on body surface (i.e., V_7_, V_8_, and V_9_) rather than conventional precordial leads, in particular V_1_, which is closer to RA (Holm et al., 1998).

The multilead index of f-wave amplitude A_ECG_ associated higher values with longer procedures, despite the poor outcome of the predictive accuracy analysis. Similar results were reported for the same parameter computed from the extended 15-lead ECG. This finding may appear in contrast with our clinical intuition, which correlates higher atrial amplitude with a more homogeneous and organized wavefront of tissue depolarization. However, similar evidence was also found in Zeemering et al. (2017), pointing out that higher f-wave amplitude may predict AF recurrence after pharmacological cardioversion. Due to the difficult interpretability of the physiological background, such aspects deserve more detailed investigation.

### Assessment of AF ablation impact in a multivariate framework

The ability of clinical parameters alone to predict CA effectiveness and predict larger CA interventions (in terms of procedural time and targets) was overall limited, in line with previous research (Lankveld et al., 2016b; Zeemering et al., 2017). As pointed out in those studies, information about patient's clinical background may be incomplete or imprecise. Parameters such as AF duration may be difficult to evaluate in some patients due to the asymptomatic or slightly symptomatic nature of some AF episodes (Lankveld et al., 2016b), thus justifying the need for the introduction of more objective, quantitative indices which can be noninvasively quantified from body surface cardiac signals. Moreover, while clinical parameters from patient's history can give an overview of AF disease severity before CA, they cannot offer any additional information during the procedure itself, e.g., between two consecutive sets of lesions, or before/after pulmonary vein isolation (unpublished data). By contrast, BSPMs can be acquired at any moment of the intervention, thus enabling a more flexible and dynamic re-evaluation of body surface AF organization and a providing a more precise indication of CA intermediate effect on arrhythmia complexity.

Characterization of protracted CA procedures was improved by the introduction of multilead f-wave amplitude, thus corroborating its ability to reflect the degree of heterogeneity of the AF wavefront propagation through the underlying atrial substrate (De Groot et al., 2010). Similarly, single-lead surface CL in V_1_ also contributed to increase classification accuracy, thus confirming that the degree of complexity of endocardial atrial activation during AF can be reflected on body surface potentials (Matsuo et al., 2009) and may require longer CA interventions to organize the arrhythmia. NDI assessed in the extended 15-lead ECG, but not in the entire BSPM lead set, equally helped improving the classification performance of clinical parameters, suggesting that in this framework the correlation between body surface AF organization and the duration of the CA procedure may come from specific anatomical locations only.

CA interventions requiring a higher number of lesions were also more accurately described in a multidimensional classification framework when NDI was integrated with patient's clinical characteristics, as confirmed by the ROC analysis. This result may be explained by the presence of a higher number of AF driving sources, located in multiple atrial locations (Lim et al., 2017), thus requiring the operator to target a higher number of atrial regions to terminate AF. Similar evidence was reported for the AF CL in V_1_, hinting at a relation between AF firing rate and the extent of its spatial distribution over atrial tissue.

CA outcome prediction was improved by combining clinical data with NDI, thus linking body surface AF organization as estimated by our marker with ablation therapy effectiveness. The results obtained were comparable with those reported in previous studies (Lankveld et al., 2016b; Zeemering et al., 2017) and underlined the relevance of body surface complexity as a marker of ablation therapy impact. Also f-wave amplitude contributed to increase multidimensional classification accuracy, as proved elsewhere (Lankveld et al., 2016b; Zeemering et al., 2017).

Surprisingly, even though we observed some improvements in classification performance as quantified by ROC analysis and we obtained results similar to those shown in other studies, the changes observed when combining clinical and signal features overall were not statistically significant according to the NRI analysis. No benefit was provided by the integration of information related to body surface AF organization, regardless of the descriptor chosen. This issue may originate from multiple factors. First, it may be due to the choice of the classification model, which may be not appropriate for our dataset, therefore other classifiers should be investigated in future works. Secondly, the accuracy of some multidimensional predictors may have been limited by the reduced number of training observations in relation to the classifier's dimension, in particular when dealing with the analysis of CA duration or with missing feature values, which could have led to biased estimates. Furthermore, the absence of improvements in classification accuracy (at least with regard to the NRI analysis) may be explained by an inappropriate selection of AF organization markers. In Zeemering et al. (2017), indices of f-wave amplitude and DF estimated in specific ECG leads were automatically selected via elastic net regularization and combined with patient's weight and right atrial volume. These results suggest that: (1) more than one signal feature may be required to better characterize the descriptive power of AF complexity; (2) contributions from specific BSPM leads (or subsets of leads) may be more relevant to the classification model than those provided by other electrodes. On the other hand, these models may have included parameters whose physiological interpretation may be less clear. For instance, in Lankveld et al. (2016b), simultaneous analysis of AF duration and single-lead f-wave amplitude (in V_6_) was predictive of CA outcome. However, lead V_1_ usually exhibits the maximum ratio of atrial to ventricular amplitude (Petrutiu et al., 2006), therefore those findings are more difficult to justify and apply to a real clinical scenario. Finally, the NRI metric itself may not be suitable for the comparison between two classification models, especially if they do not fit the training datasets accurately (Pepe et al., 2015). Furthermore, since the assessment of NRI significance proposed by Pencina et al. (2008) has never been systematically validated (Kerr et al., 2014), further metrics should be investigated to validate NRI results.

### Limitations and perspectives

The diversity of the criteria used for AF complexity definition and clinical CA protocols and endpoints made the comparison between parameters from current literature more challenging. While a more systematic overview of classical descriptors of AF spatiotemporal organization has been attempted (Bonizzi et al., 2014; Lankveld et al., 2014), the integration of such contributions to clinical practice is still an open issue, and the predictive accuracy of most of the univariate indices examined needs to be improved.

To this end, we tested whether our understanding of AF characteristics and therapy management could benefit from combining patient's clinical characteristics and signal complexity features. However, as pointed out in section Assessment of AF Ablation Impact in a Multivariate Framework, the evaluation of multidimensional classifiers may be limited by several factors, including the limited number of observations, the choice of the classification model and the complexity indices, and the metrics used for model comparison. Even though our study offers some relevant insights into AF multidimensional analysis, several aspects should be investigated with more attention in future works, in particular the type of signal features and the BSPM leads to be selected, potentially through automatic algorithms, as well as the introduction of information coming from other imaging systems, such as fibrosis distribution assessed by magnetic resonance (Jadidi et al., 2013).

The correlation between the examined indices of AF pathophysiology and impact of the CA strategy and AF duration could not be significantly quantified by a pairwise Pearson's linear analysis neither in our study nor by other groups (Ammar et al., 2014). This limitation also justifies the choice to discriminate between persistent and long-lasting AF patients according to the definition provided in Calkins et al. (2012) and regard AF duration as a dichotomous variable rather than continuous, since none of the descriptors of AF organization linearly correlated with this clinical parameter.

Secondly, frequency measures of AF organization were not explored in our comparative analysis, due to the impossibility to retrieve the original BSPMs from the acquisition system, as TQ interval segmentation is performed at the moment of the ablation procedure. To this end, we examined the AF CL in lead V_1_, which was demonstrated to correlate with the intracardiac atrial fibrillatory rate in Matsuo et al. (2009). While in that study this measure was manually assessed in standard ECG, in our work we introduced an algorithm for the automatic computation of the rate of atrial signal local extrema, which may be sensitive to the presence of artifacts and spurious peaks if proper settings as those described in section Comparison With Other Descriptors of AF Complexity on Surface Recordings are not applied.

Some BSPM electrodes may present artifacts due to patient's breathing or mechanical motion. As a consequence, all signals have been visually inspected and electrodes with too high levels of noise were discarded.

The assumption that more complex AF forms require the operator to target a higher number of atrial sites is supported by previous clinical studies claiming that in advanced AF forms the density of driving sources over atrial tissue tends to be higher, thus covering more sites (Lim et al., 2017). However, this should be confirmed by phase mapping analysis as well.

The ability of the AF complexity parameters to predict long-term CA outcome has not been investigated due to the unavailability of such information for some patients at the moment of the study, and it therefore represents an open perspective of this research.

Furthermore, it may be clinically relevant to assess changes in complexity in BSPMs within the CA procedure and between intermediate steps (for instance, after pulmonary vein isolation), so as to understand whether modifications of atrial substrate by CA immediately reflect on surface electrical activity.

Future research also includes the investigation of the relation between body surface complexity and AF termination sites. This task may present some challenges, in particular in relation to the identification of the most suitable electrodes to be associated with the atrial regions of interest.

Finally, the application of our noninvasive methodology to other types of AF therapy (e.g., electrical cardioversion) may help improve their management.

## Conclusions

This research put forward a tool for the quantification of AF organization by PCA of multilead BSPMs. Our analysis underlined a significant correlation of such noninvasive information with AF chronification and CA practice. This methodology can provide relevant insights into AF substrate characterization from the body surface ablation therapy.

## Author contributions

All authors have made substantial contributions to this study. MM designed the study, implemented the signal processing algorithms, analyzed, and interpreted the results, and drafted the manuscript. TP, ND, and JD performed CA procedures and contributed to the interpretation of the clinical data. CD-P and SP acquired and segmented atrial activity signals from BSPM recordings. PJ, MéH, and MiH supervised clinical data acquisition and helped assessing signal processing algorithm performance. RD helped conceive the study, provided feedback about the implementation of the methods and the interpretation of the results, and revised the manuscript.

### Conflict of interest statement

The authors declare that the research was conducted in the absence of any commercial or financial relationships that could be construed as a potential conflict of interest.

## Abbreviations

AAD: antiarrhythmic drugs
AF: atrial fibrillation
ANOVA: one-way analysis of variance
AUC: area under curve
BSPM: body surface potential map
CA: catheter ablation
CL: cycle length
DCC: electrical cardioversion
DF: dominant frequency
ECG: electrocardiogram
EGM: electrogram
LA: left atrium
LAA: left atrial appendage
LR: logistic regression
NA: not applicable
NDI: nondipolar component index
NMSE: normalized mean square error
NRI: net reclassification improvement
ns: not significant
PCA: principal component analysis
RA: right atrium
ROC: receiver operating characteristic
std: standard deviation.
